# Chinese culture became more individualistic: Evidence from family structure, 1953-2017

**DOI:** 10.12688/f1000research.128448.2

**Published:** 2023-04-25

**Authors:** Yuji Ogihara

**Affiliations:** 1 Faculty of Science Division II, Tokyo University of Science, Tokyo, 162-8601, Japan; 2 Department of Psychology, University of California, Los Angeles, Los Angeles, 90095-1563, USA; 3 Department of Cognitive Psychology in Education, Graduate School of Education, Kyoto University, Kyoto, 606-8501, Japan

**Keywords:** individualism, family, cultural change, China, temporal change, divorce, household, family structure

## Abstract

Previous research has indicated that some aspects of Chinese culture became more individualistic. However, prior studies have suggested a decrease in individualism in other aspects of China. Thus, it was unclear whether China became more individualistic. Therefore, the current research investigated whether Chinese culture became more individualistic by examining historical changes in family structure. Specifically, I analyzed temporal shifts in the divorce rate and household size, which have been confirmed as valid representative indicators of individualism. Results showed that the divorce rate increased between 1978 and 2017 and household size decreased between 1953 and 2017, indicating a rise in individualism. Moreover, analyses suggested that the one-child policy was unlikely the sole and major factor in the decrease in household size. Additionally, the aggregated score of divorce rate and household size demonstrated a clear increase in individualism. Therefore, the present research provided further evidence of the rise in individualism in China.

## Introduction

### Increase in individualism in China

Previous research has indicated that Chinese culture has become more individualistic over time. Taras, Steel, and Kirkman (2012) conducted a meta-analysis of studies that used Hofstede (1980)’s cultural framework (four dimensions; individualism, power distance, masculinity, uncertainty avoidance). In this research, they found that individuals in China came to hold more individualistic values from the 1980s to the 2000s, which shows that Chinese culture became more individualistic at the individual level.

Not only at the individual level, but also at the cultural (group) level, Chinese culture has changed toward greater individualism. A previous study examined historical changes in individualism-collectivism in China between 1950 and 2008 ( Hamamura and Xu, 2015). It analyzed changes in the frequency of first-person singular/plural pronouns used in Chinese published books over the period by utilizing a database of millions of books published in various languages (Google Books Ngram). It found that the rate of first-person singular pronouns increased and the rate of first-person plural pronouns decreased. These results suggest an increase in individualism in China (also see, Yu et *al.*, 2016). Moreover, Zeng and Greenfield (2015) have indicated that the prevalence of words that were considered to reflect individualistic values/behaviors (e.g., “autonomy”, “choose”) increased in Chinese books published between 1970 and 2008, reflecting a shift toward greater individualism in Chinese culture.

Not only values but also behaviors may have become more individualistic. It has been claimed that unique names increased in China between 1950 and 2009, suggesting a rise in the need for uniqueness and individualism ( Cai, Zou, Feng, Liu, & Jing, 2018; Bao, Cai, Jing, & Wang, 2021; but also see, Ogihara, 2020b).

### Decrease in individualism in China

In contrast, regarding values, some studies have indicated that Chinese culture has become less individualistic. It has been reported that individuals in China came to hold less individualistic values between 1990 and 2007 ( Santos, Varnum, & Grossmann, 2017). This study demonstrates that at the individual level, China has shifted toward a less individualistic culture. ^1^

Further, Zeng and Greenfield (2015) found that, contrary to their hypothesis, the prevalence of the two words (“obliged” and “give”; out of eight words examined) that were considered to reflect collectivistic values/behaviors increased in usage in Chinese books published between 1970 and 2008. This result may show that China became less individualistic at the cultural level. ^2^

### Current research

Thus, it is unclear whether Chinese culture has become more individualistic. Particularly, there have been only two studies examining changes in the behavioral aspect in China (naming; Bao *et al.*, 2021; Cai *et al.*, 2018). Thus, it is important to examine cultural changes in China by using other behavioral measurements. Therefore, the current research investigated cultural changes in China using two other behavioral indicators of individualism that have already been validated but have not been used to examine cultural changes in China: divorce rates and household size.

Divorce rates and household size are behavioral measurements reflecting individualistic tendencies. In individualistic cultures, family structure tends to be freer and looser compared to that in collectivistic cultures (e.g., Georgas *et al.*, 2001; Triandis, 1995). This leads people to live separately and independently of other family members, contributing to higher divorce rates and smaller households.

Indeed, divorce rates and household size are correlated with the indices of individualism developed by Hofstede (1980) and Triandis in the predicted direction at the national level (e.g., Diener, Diener, & Diener, 1995; Hamamura, 2012; Lester, 1995; Toth & Kemmelmeier, 2009). Moreover, prior research has shown that both indicators are associated with variables whose relations to individualism have been conceptually and empirically confirmed, such as pronoun drop ( Kashima & Kashima, 1998) and pathogen prevalence ( Murray & Schaller, 2010) at the national level (e.g., Hamamura, 2012). ^3^ Thus, divorce rates and household size have been frequently used as indices of individualism (e.g., Diener *et al.*, 1995; Grossman & Varnum, 2015; Hamamura, 2012; Ogihara, 2018b, 2020a; Vandello & Cohen, 1999). ^4^

## Methods

### Indicators

As the measure of divorce rates, the divorce-to-marriage ratio was used. Data covered the period between 1978 and 2017 and came from the National Bureau of Statistics of China (2018). Data on household sizes covered the period between 1953 and 2017 and were drawn from the National Bureau of Statistics of China (2018).

### Aggregated score

Although divorce rates and household size have been confirmed as valid indicators of individualism, each indicator may be simultaneously influenced by factors other than individualism. For instance, celebrity divorces may impact divorce rates. By aggregating the two indicators, the random errors of each indicator cancel each other out, successfully reducing the influence of these random errors. Thus, an aggregated score was calculated by averaging the divorce rate (z-transformed) and household size (z-transformed and reversed). This strategy of analysis has been frequently used in prior research (e.g., Diener *et al.*, 1995; Grossmann & Varnum, 2015; Hamamura & Xu, 2015; Ogihara, 2018b, 2020a; Santos *et al.*, 2017; Yu *et al.*, 2016; Vandello & Cohen, 1999).

## Results

Simple Pearson’s and Kendall’s correlation coefficients among each indicator are shown in Table 1.

**Table 1.** Simple Pearson’s and Kendall’s correlation coefficients among year and indicators of individualism.

	Year	Divorce rate	Household size	Aggregated score
Year	—	.94	-.90	.99
Divorce rate ( *N* = 40; 1978-2017)	.95	—	-.85	.96
Household size ( *N* = 33; 1953-2017)	-.86	-.85	—	-.96
Aggregated score ( *N* = 31; 1978-2017)	.94	.96	-.89	—

*Note.* Scores in the upper half indicate Pearson’s correlation coefficients, while those in the lower half represent Kendall’s correlation coefficients. Both results were consistent with each other. The aggregated score was calculated by averaging the divorce rate (z-transformed) and household size (z-transformed and reversed).

### Divorce rate

**Figure 1.**
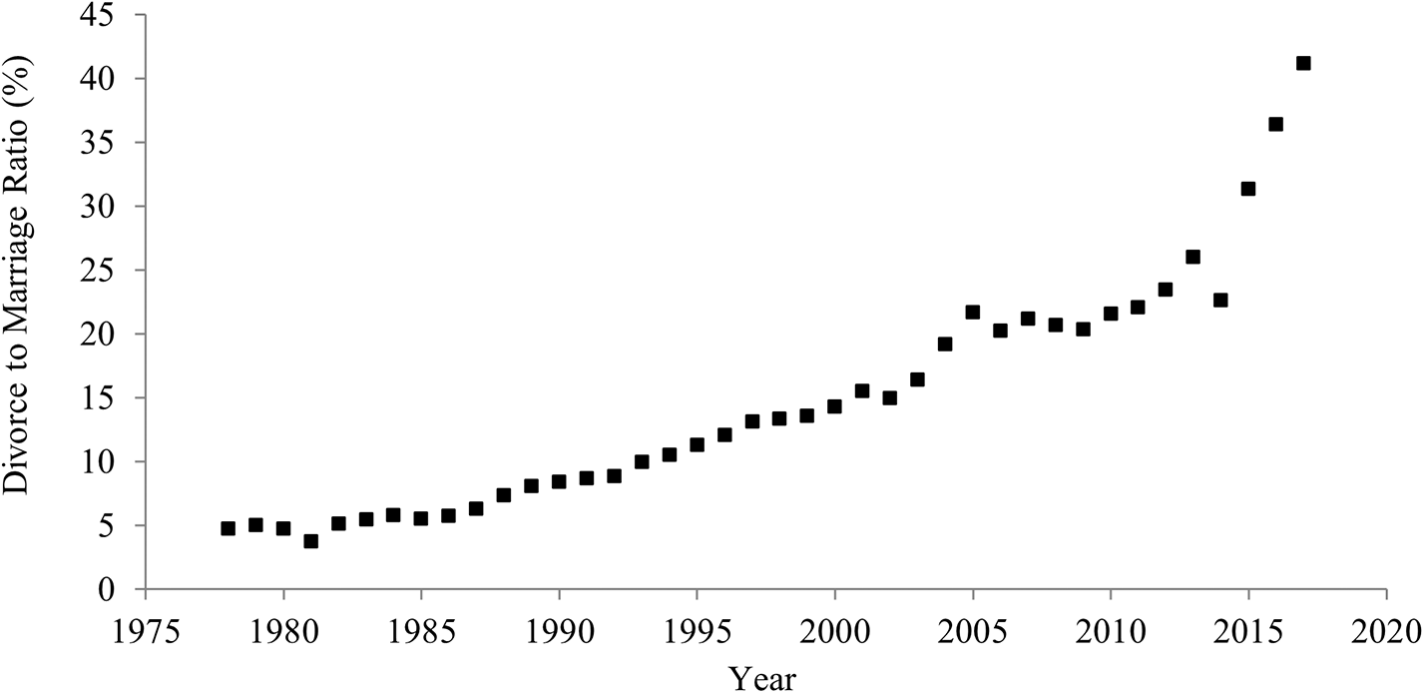
Divorce rate in China, 1978-2017.

Figure 1 shows historical shifts in the divorce rate between 1978 and 2017 in China. The divorce rate significantly rose over the last 40 years. In 1978, 4.8 out of 100 couples divorced, but in 2017, 41.1 out of 100 couples experienced a divorce. The correlation between the year and the divorce rate was strongly positive ( Table 1), suggesting an increase in individualism.

### Household size

**Figure 2.**
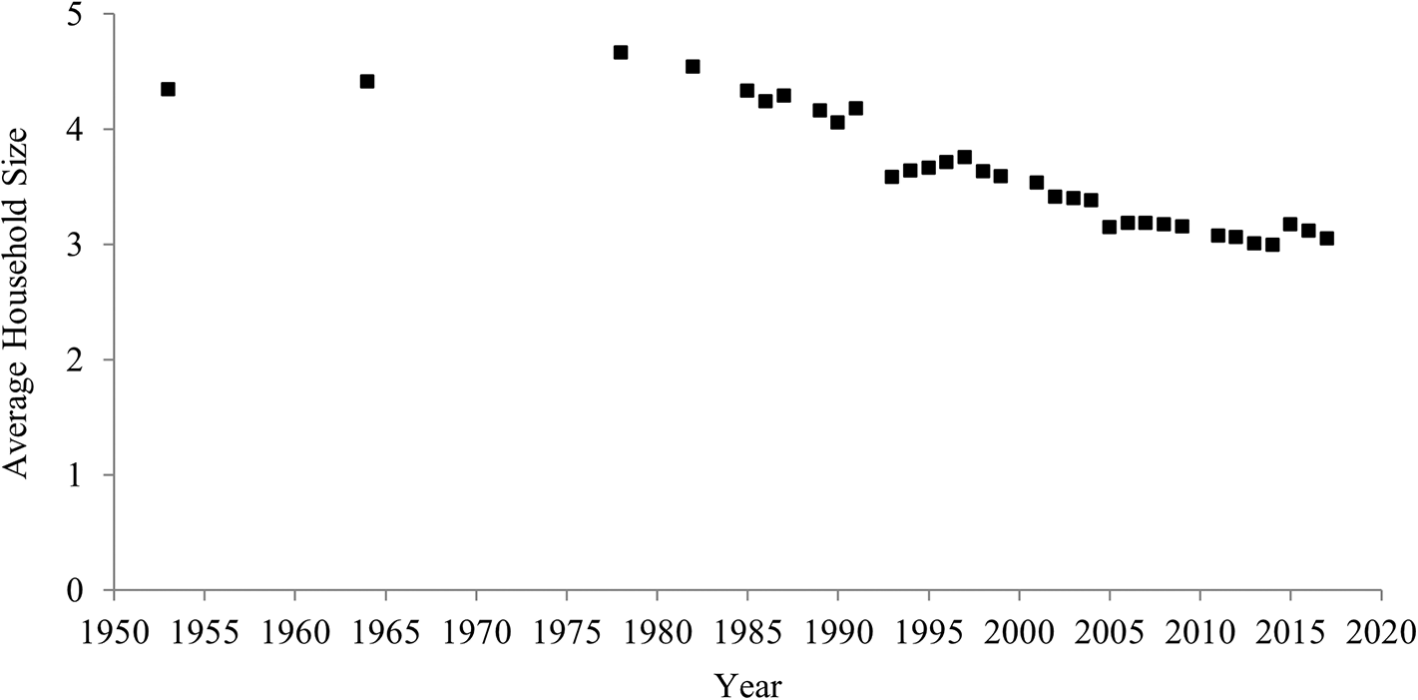
Average household size in China, 1953-2017.

Figure 2 indicates temporal shifts in household size between 1953 and 2017 in China. Household size declined over the past 60 years. In 1953, the average household consisted of 4.3 people, but in 2017 the average was 3.1 people. The correlation between year and household size was highly negative ( Table 1), which indicates a rise in individualism.

**The one-child policy and household size.** One might expect that the decrease in the average household size was caused solely by the one-child policy and may thus not reflect an increase in individualism. China’s one-child policy, which was introduced in 1980 and ended in 2015, penalized parents for having more than one child. This policy may have decreased the birth rate, in turn decreasing the average household size.

**Figure 3.**
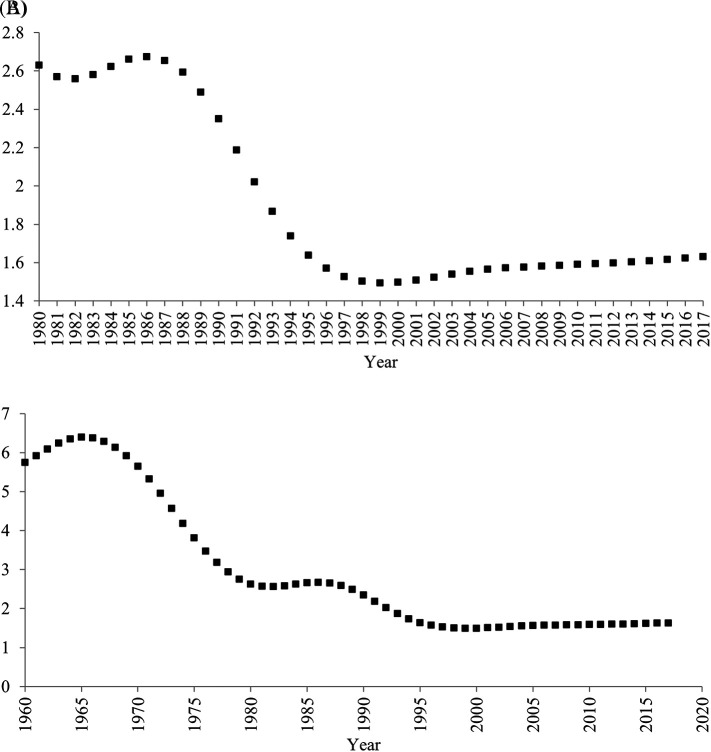
Total Fertility Rate (births per woman) in China, 1960-2017. *Note.* Data were drawn from the World Bank (2019). (A) 1980-2017 (B) 1960-2017.

However, it is difficult to assert that the decrease in the average household size was caused solely by the one-child policy for three reasons. First, it is not necessarily correct that the one-child policy continued to decrease the birth rate ( Whyte, Feng, & Cai, 2015). Indeed, after the one-child policy was introduced in 1980, the fertility rate did not continue to decrease ( Figure 3A). Contrary to a common myth, even after the implementation of the one-child policy, the fertility rate increased between 1983 and 1986, and between 2000 and 2017. For more than half period of time (22 years; 1983-1986, 2000-2017) when the one-child policy was introduced (36 years; 1980-2015), the fertility rate did not decrease.

Second, it is not always correct that decreases in birth rate lead to decreases in household size. Indeed, for these periods when the fertility rate increased (1983-1986, 2000-2017), the household size continued to decrease. Moreover, the fertility rate remarkably decreased before, rather than after, the one-child policy was adopted in 1980 ( Figure 3B). In 1960, approximately six babies were born to one woman, whereas in 1979 approximately three babies were born to one woman. Even during this period when the fertility rate remarkably decreased, the average household size did not seem to decrease.

Third, even after the one-child policy ended in 2015, the average household size continued to decrease. If the one-child policy had a strong influence on household size, after the policy ended, household size should have increased (but it decreased).

Thus, although the one-child policy may have contributed to the decrease in household size to some extent, its effect was not large. Therefore, the one-child policy was unlikely to be the sole and major factor in this decrease in the average household size.

### Aggregated score

**Figure 4.**
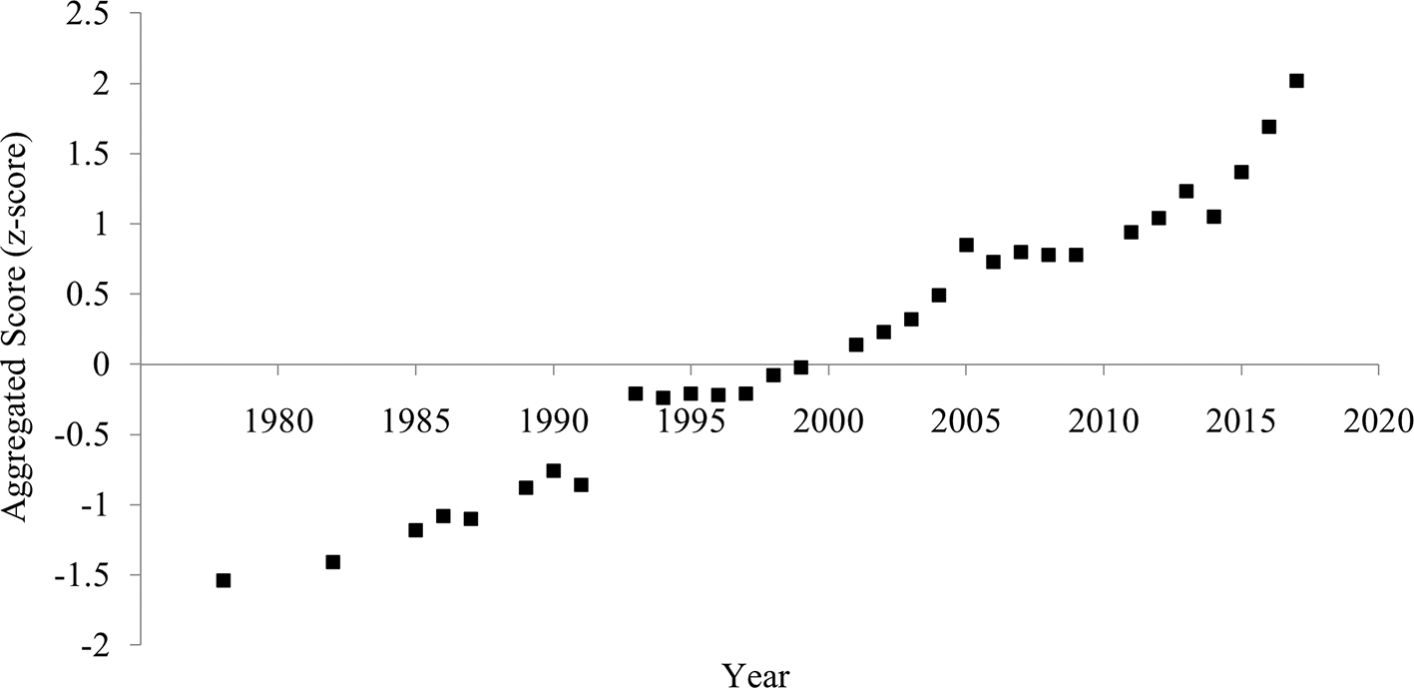
Aggregated score of individualism in China, 1978-2017. *Note.* The aggregated score was calculated by averaging the divorce rate (z-transformed) and household size (z-transformed and reversed).

The aggregated score of divorce rate and household size showed a clearer and more consistent pattern of increased individualism than each indicator ( Figure 4). The correlation between the year and the aggregated score was strongly positive ( Table 1), suggesting an increase in individualism.

### Crude divorce rate

**Figure 5.**
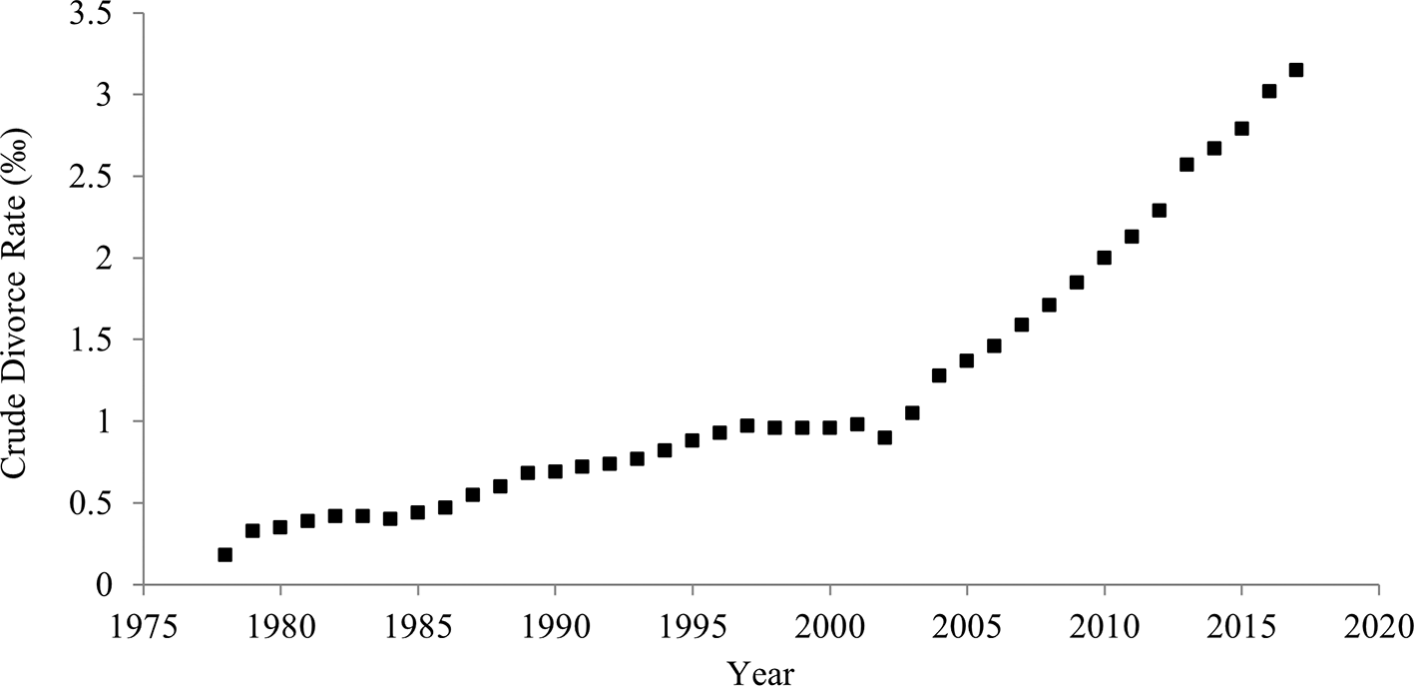
Crude divorce rate in China, 1978-2017.

**Figure 6.**
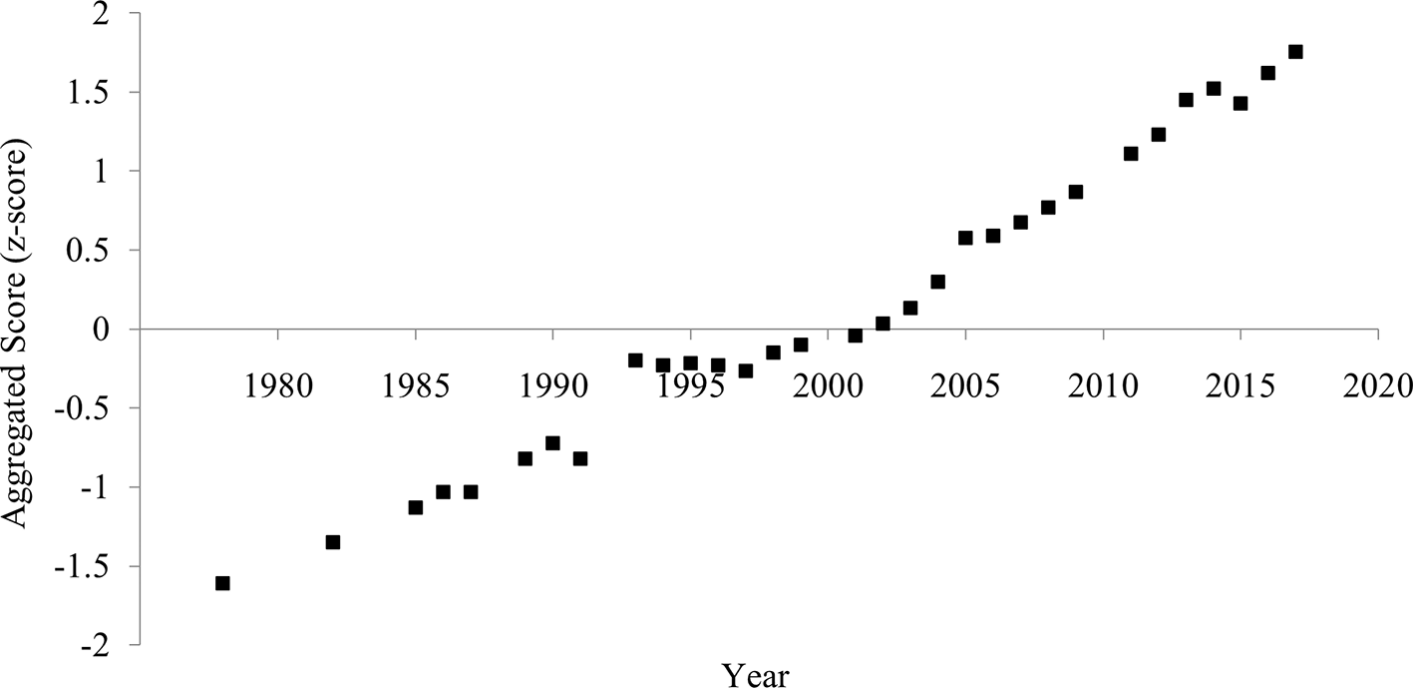
Aggregated score of individualism in China, 1978-2017. *Note.* The aggregated score was calculated by averaging the crude divorce rate (z-transformed) and household size (z-transformed and reversed).

When the crude divorce rate (rate per 1,000 persons) was used instead of divorce-to-marriage ratio, the results were unchanged ( Table 2; Figure 5; Figure 6). The Pearson’s and Kendall’s correlation coefficients between crude divorce rate and divorce-to-marriage ratio were .95 and .92, respectively.

**Table 2.** Simple Pearson’s and Kendall’s correlation coefficients among year and indicators of individualism (crude divorce rate).

	Year	Crude divorce rate	Household size	Aggregated score
Year	—	.93	-.90	.99
Crude divorce rate ( *N* = 40; 1978-2017)	.97	—	-.83	.96
Household size ( *N* = 33; 1953-2017)	-.86	-.84	—	-.95
Aggregated score ( *N* = 31; 1978-2017)	.95	.92	-.92	—

*Note.* Scores in the upper half indicate Pearson’s correlation coefficients, while those in the lower half represent Kendall’s correlation coefficients. Both results were consistent with each other. The aggregated score was calculated by averaging the crude divorce rate (z-transformed) and household size (z-transformed and reversed).

## Discussion

The current research showed that Chinese culture became more individualistic over the past 60 years by using behavioral indicators that had not been sufficiently examined. Past research showed the rise in individualism in China ( Bao *et al.*, 2021; Cai *et al.*, 2018; Hamamura & Xu, 2015; Taras *et al.*, 2012; Yu *et al.*, 2016; Zeng & Greenfield, 2015). However, some studies indicated the fall of individualism in China ( Santos *et al.*, 2017; Zeng & Greenfield, 2015). Thus, it remained unclear whether Chinese culture became more individualistic. Particularly, it was unclear whether China shifted toward greater individualism in the behavioral aspect. The present research investigated temporal changes in the two behavioral indicators (divorce rate and household size), which have been used to examine cultural changes in other countries (e.g., Hamamura, 2012; Grossmann & Varnum, 2015; Ogihara, 2018b, 2020a).

Results showed that the divorce rate increased dramatically between 1978 and 2017, and that household size steadily shrank between 1953 and 2017, suggesting an increase in individualism in China. Further, analyses indicated that the one-child policy was not the major factor of the decrease in household size. Moreover, the aggregated score of divorce rate and household size demonstrated a clearer pattern of the increase in individualism than each indicator. Therefore, this research contributes to the accumulation of the literature demonstrating the rise in individualism in China by using different behavioral indicators of individualism.

### Similar phenomena in Japan

It is more likely that these mixed changes are found in historically collectivistic cultures that are becoming more individualistic. Indeed, such changes were also found in Japan, another country in East Asia (for a review, see Ogihara, 2017, 2018a).

On the one hand, people in Japan came to live more independently from other family members ( Hamamura, 2012; Ogihara, 2018b), increasingly gave more unique names to their babies ( Ogihara, 2015, 2021a, 2021b; Ogihara *et al.*, 2015), and came to hold more individualistic values ( Hamamura, 2012; Taras *et al.*, 2012), indicating an increase in individualism. On the other hand, a decrease in and non-change of individualistic values have been reported ( Hamamura, 2012).

It is suggested that these mixed changes may be related to difficulties in adapting to a new environment (e.g., Ogihara, 2016; Ogihara & Uchida, 2014; Ogihara *et al.*, 2014, 2016). Still, it is difficult to say that there is a sufficient amount of research on how cultures change and how people adapt to such cultural changes in East Asia. Therefore, it is important to investigate historical changes in these cultures in more detail.

### Limitations and future directions

The current research used family structure (divorce rate and household size) as representative indicators of individualism. Nevertheless, it is still unclear whether other aspects of individualism show the same trend. To examine cultural changes in China, it is necessary to investigate various aspects of cultural changes by analyzing different indicators from a broad set of perspectives.

Although the present research demonstrated that Chinese culture became more individualistic, it is not clear why this change arose. There are many possible factors to cause this change (e.g., economic wealth, social mobility, subsistence styles, ecological/societal threats, and urbanization). Future studies should answer this question.

## Data Availability

Data used in this study are available from the National Bureau of Statistics of China (2018) website ( http://www.stats.gov.cn/english/).

## Footnotes

^1^
Although this study described global trends of temporal changes in individualism, it has some limitations. For example, the authors aggregated three items from the World Values Survey to examine temporal shifts in individualistic values, but the inter-item correlations were not high (.11 < τs < .24; ordinal-level Kendall’s τ), possibly implying that the validity of these items may be low. Moreover, as the authors already noted in their article, the data points for China were only four (1990, 1995, 2001, and 2007), and they covered a relatively short period of time (17 years). This limited number of data points might have led to a failure to detect actual historical changes in individualistic values in China. These limitations may be related to the inconsistency of the findings. Indeed, their study showed that most (39 out of 53) of the countries they examined indicated a substantial increase in individualistic values, whereas only five countries (China, Armenia, Croatia, Ukraine, and Uruguay) exhibited a nonnegligible decrease in individualistic values.

^2^
However, these words increased in frequency only slightly. As the authors emphasized in their article, the contrasting individualistic words (“choose” and “get”) that were analyzed increased more remarkably (for the details of their interpretation, see Zeng and Greenfield, 2015).

^3^
Although the validities of the two indicators have been confirmed at the national level, it is desirable to check the validities at the area (e.g., provincial) level in China.

^4^
In some studies, other socio-demographic variables were used as indicators representing individualism such as percentage of self-employed people (e.g., Vandello & Cohen, 1999) and the ratio of single-child families relative to multi-child families (e.g., Grossmann & Varnum, 2015). Although their conceptual associations with individualism are understandable, their empirical relations with individualism are not sufficiently clear so far. Thus, in this research, divorce rates and household sizes, which have already been empirically confirmed to be valid indicators of individualism (e.g., Hamamura, 2012; Grossmann & Varnum, 2015), were used.
