# Supplementary material for: Chinese culture became more individualistic: Evidence from family structure, 1953-2017
Source: F1000Res. 2023 May 25;12:10. Originally published 2023 Jan 4. [Version 3] doi: 10.12688/f1000research.128448.3 (PMC10355225; doi:10.12688/f1000research.128448.3)
Supplement: Peer review history

# Peer review: Reviewer response for version 3

**DOI:** 10.5256/f1000research.148523.r175366

**Published:** 2023-06-21

**Authors:** Eunsoo Choi

**Affiliations:** 1 School of Psychology, Korea University, Seoul, South Korea

The author has addressed all my comments.

Is the work clearly and accurately presented and does it cite the current literature?

Yes

If applicable, is the statistical analysis and its interpretation appropriate?

I cannot comment. A qualified statistician is required.

Are all the source data underlying the results available to ensure full reproducibility?

Yes

Is the study design appropriate and is the work technically sound?

Yes

Are the conclusions drawn adequately supported by the results?

Yes

Are sufficient details of methods and analysis provided to allow replication by others?

Partly

Reviewer Expertise:

NA

I confirm that I have read this submission and believe that I have an appropriate level of expertise to confirm that it is of an acceptable scientific standard.

# Peer review: Reviewer response for version 2

**DOI:** 10.5256/f1000research.146975.r158977

**Published:** 2023-05-09

**Authors:** Eunsoo Choi

**Affiliations:** 1 School of Psychology, Korea University, Seoul, South Korea

The present research looked at the changes in divorce rates and household size in China as indices measuring individualistic behaviors. The results showed that aggregated score of combining divorce rates and household size steadily increased over the past 40 years in China, indicating that the individualism increased in this culture.

 This research demonstrates the changes in these two indices that are known to be related with individualism of the society.

 There are a couple of minor comments on this brief report:  

- Table 1 is missing which parts (above and below) of the diagonal are about Pearson's correlation and Kendall's correlation coefficients.
- As a more comprehensive test, the crude divorce rate is used in addition to the divorce-to-marriage ratio. Please clarify how crude divorce rate is calculated. Are they the ratio of individuals who got divorced amongst 1,000 married people or people in general? 
- As this study is looking at social structural change in China, which I agree is intricately related with the cultural values of the members of the society, it would be important not to overgeneralize the finding to changes in individualism. It is possible that there is a mismatch between individuals' cultural values and societal structure (household size, in particular), therefore, it should be clearly noted that the cultural values systems of individuals may not have caught up with the changes in the social system as indicated by the increased divorced rates & decreased household size.
- The author argues that the reason why the one-child policy might not have driven the decrease in household size is due to the disconnect between the policy and birthrate. Then, additional explanation for why the household size decreased may render insight into the changes in culture in China. Is the creased household size due to decreased households living with grandparents or an increase in one-person household? Some interpretation of this may be helpful.

Is the work clearly and accurately presented and does it cite the current literature?

Yes

If applicable, is the statistical analysis and its interpretation appropriate?

I cannot comment. A qualified statistician is required.

Are all the source data underlying the results available to ensure full reproducibility?

Yes

Is the study design appropriate and is the work technically sound?

Yes

Are the conclusions drawn adequately supported by the results?

Yes

Are sufficient details of methods and analysis provided to allow replication by others?

Partly

Reviewer Expertise:

Emotion, well-being, culture

I confirm that I have read this submission and believe that I have an appropriate level of expertise to confirm that it is of an acceptable scientific standard, however I have significant reservations, as outlined above.

# Response

**Published:** 2023-05-20

**Authors:** Yuji Ogihara

**Affiliations:** Psychology, Aoyama Gakuin Daigaku, Shibuya, Tokyo, Japan

May 20th, 2023

 Dear Dr. Eunsoo Choi,

 Thank you very much for reviewing my manuscript and providing valuable comments.

 I have modified the manuscript according to your comments. I offer my responses to each comment below.

 I have copied and pasted all of your comments without making changes.

*The present research looked at the changes in divorce rates and household size in China as indices measuring individualistic behaviors. The results showed that aggregated score of combining divorce rates and household size steadily increased over the past 40 years in China, indicating that the individualism increased in this culture.*

*This research demonstrates the changes in these two indices that are known to be related with individualism of the society.*

*There are a couple of minor comments on this brief report:*

*Table 1 is missing which parts (above and below) of the diagonal are about Pearson's correlation and Kendall's correlation coefficients.*

 Thank you for your helpful comment. The PDF and the submitted manuscript of this article have a note of Table 1 (“Note. Scores in the upper half indicate Pearson’s correlation coefficients, while those in the lower half represent Kendall’s correlation coefficients. Both results were consistent with each other. The aggregated score was calculated by averaging the divorce rate (z-transformed) and household size (z-transformed and reversed).”) However, as you pointed out, this note is missing on the webpage of this article. I asked the F1000Research office to modify this editorial error. 

*As a more comprehensive test, the crude divorce rate is used in addition to the divorce-to-marriage ratio. Please clarify how crude divorce rate is calculated. Are they the ratio of individuals who got divorced amongst 1,000 married people or people in general?*

 I appreciate your suggestion. Following your suggestion, I have added an explanation of the crude divorce rate as below. It is the rate per 1,000 persons in general, not limited to married people.

 “When the crude divorce rate (rate per 1,000 persons in general, not limited to married people) was used instead of divorce-to-marriage ratio, the results were unchanged (Table 2; Figure 5; Figure 6).”

*As this study is looking at social structural change in China, which I agree is intricately related with the cultural values of the members of the society, it would be important not to overgeneralize the finding to changes in individualism. It is possible that there is a mismatch between individuals' cultural values and societal structure (household size, in particular), therefore, it should be clearly noted that the cultural values systems of individuals may not have caught up with the changes in the social system as indicated by the increased divorced rates & decreased household size.*

 Thank you for your comment. In fact, I discuss these complex changes in individualism, which includes not only values but also other aspects such as behaviors and cultural products, in China (and Japan) in this paper. The indices I used in this paper (i.e., divorce rate and household size) have been confirmed to be valid indicators of individualism and show the changes in some aspects of individualism. Thus, I explain this point as a limitation in the Limitations and future directions section as below. If you think this is insufficient, I would appreciate it if you could specify which part is insufficient.

 “The current research used family structure (divorce rate and household size) as representative indicators of individualism. Nevertheless, it is still unclear whether other aspects of individualism show the same trend. To examine cultural changes in China, it is necessary to investigate various aspects of cultural changes by analyzing different indicators from a broad set of perspectives.”

*The author argues that the reason why the one-child policy might not have driven the decrease in household size is due to the disconnect between the policy and birthrate. Then, additional explanation for why the household size decreased may render insight into the changes in culture in China. Is the creased household size due to decreased households living with grandparents or an increase in one-person household? Some interpretation of this may be helpful.*

 I appreciate your constructive suggestion. Following your suggestion, I have added a possible explanation of why the household size decreased as below.

 “Many factors could explain this decrease in household size (e.g., an increase in people living alone, a decrease in households with multiple generations).”

 Thank you for your further consideration of this manuscript.

 I look forward to hearing from you at your earliest convenience.

 Sincerely,

 Yuji Ogihara, Ph.D.

 Department of Psychology, College of Education, Psychology and Human Studies, Aoyama Gakuin University

 Address: 4-4-25 Shibuya, Shibuya-ku, Tokyo, 150-8366, Japan

 E-mail: yogihara@ephs.aoyama.ac.jp

Web: https://sites.google.com/site/yujiogiharaweb/english

# Peer review: Reviewer response for version 2

**DOI:** 10.5256/f1000research.146975.r170836

**Published:** 2023-04-25

**Authors:** Han-Wu-Shuang Bao

**Affiliations:** 1 Manchester China Institute, The University of Manchester, Manchester, UK

Thanks for the author's revised version that has properly addressed my concerns. Regarding the first point about adding more data points, I agree with the author that this is less critical to the main purpose of the article. The currently tested year range has already provided convincing evidence. As a further suggestion that the author may consider in future studies, it will be meaningful to test a longer time period and conduct a time-series analysis (e.g., Granger causality test) to examine the socio-ecological causes and/or consequences of the increasing individualism in China. Overall, I have no further comments to make on the current version of this article.

Is the work clearly and accurately presented and does it cite the current literature?

Yes

If applicable, is the statistical analysis and its interpretation appropriate?

Yes

Are all the source data underlying the results available to ensure full reproducibility?

Yes

Is the study design appropriate and is the work technically sound?

Partly

Are the conclusions drawn adequately supported by the results?

Yes

Are sufficient details of methods and analysis provided to allow replication by others?

Yes

Reviewer Expertise:

Cultural psychology, Cultural change, Individualism, Name, China

I confirm that I have read this submission and believe that I have an appropriate level of expertise to confirm that it is of an acceptable scientific standard.

# Peer review: Reviewer response for version 1

**DOI:** 10.5256/f1000research.141039.r158975

**Published:** 2023-01-13

**Authors:** Han-Wu-Shuang Bao

**Affiliations:** 1 Manchester China Institute, The University of Manchester, Manchester, UK

In this article, the author summarized previous contradictory findings on the changes in individualism in China over the past decades, and provided further evidence from two social indicators (i.e., divorce rate and household size) for the increasing individualism in China. While data on such indicators are publicly available and quite easy to be collected from the National Bureau of Statistics of China ( data.stats.gov.cn), it was indeed surprising that to date no studies have reported these indicators as evidence for cultural changes in China. Thus, the author’s current brief report would be necessary for a more complete understanding of Chinese cultural changes.

 Nonetheless, I have some suggestions for the author to further improve this article, and also expect a revised version of the manuscript from the author.

1. The author collected the data in 2018, which covered the period only until 2017. This made the results somewhat out of date. Based on my own search ( data.stats.gov.cn), it is certain that now the National Bureau of Statistics of China has updated the data until 2021. Therefore, I strongly suggest the author collecting more data points (i.e., 2018, 2019, 2020, and 2021) to provide the latest evidence. This would strengthen the potential impact of this article, and also help address the concern about the one-child policy because more data points (than merely 2016 and 2017) could be tested after the policy’s end in 2015 (i.e., to check whether household size continued to decrease even when Chinese parents are encouraged to give birth to more than one child since 2015).
2. It would be better to disclose the limitations of previous findings, which would help readers better evaluate different pieces of evidence. For example, in Santos et al.’s (2017) study, they only had 4 data points for China (1990, 1995, 2001, 2007) on three self-report items of cultural values ^1^. This could be main limitations of this specific finding and would make the idea that “individualism is decreasing in China” less convincing. The author may provide further critical comments when summarizing the literature.
3. Since Bao et al. (2021) has provided more valid evidence for the increasing name uniqueness in China ^2^ and has addressed Ogihara’s (2020) ^3^ concerns on Cai et al. (2018) ^4^, it is necessary to also cite Bao et al. (2021) ^2^ when citing Cai et al. (2018) ^4^ in this article:
  1. P. 2 (of the pdf version of the article) “Particularly, there has been only one study examining changes in the behavioral aspect in China (naming; Cai et al., 2018).” should be “Particularly, there has been **two** studies examining changes in the behavioral aspect in China (naming; **Bao et al., 2021**; Cai et al., 2018).”
  2. P. 8 “Past research showed the rise in individualism in China ( **Bao et al., 2021**; Cai et al., 2018; …)”.

Is the work clearly and accurately presented and does it cite the current literature?

Yes

If applicable, is the statistical analysis and its interpretation appropriate?

Yes

Are all the source data underlying the results available to ensure full reproducibility?

Yes

Is the study design appropriate and is the work technically sound?

Partly

Are the conclusions drawn adequately supported by the results?

Yes

Are sufficient details of methods and analysis provided to allow replication by others?

Yes

Reviewer Expertise:

Cultural psychology, Cultural change, Individualism, Name, China

I confirm that I have read this submission and believe that I have an appropriate level of expertise to confirm that it is of an acceptable scientific standard, however I have significant reservations, as outlined above.

## References

[1] : Global Increases in Individualism. *Psychol Sci* .2017;28(9) : 10.1177/0956797617700622 1228-1239 10.1177/0956797617700622 28703638

[2] : Novel Evidence for the Increasing Prevalence of Unique Names in China: A Reply to Ogihara. *Front Psychol* .2021;12: 10.3389/fpsyg.2021.731244 731244 10.3389/fpsyg.2021.731244 34938229PMC8685573

[3] : Unique Names in China: Insights From Research in Japan-Commentary: Increasing Need for Uniqueness in Contemporary China: Empirical Evidence. *Front Psychol* .2020;11: 10.3389/fpsyg.2020.02136 2136 10.3389/fpsyg.2020.02136 33117207PMC7550777

[4] : Increasing Need for Uniqueness in Contemporary China: Empirical Evidence. *Front Psychol* .2018;9: 10.3389/fpsyg.2018.00554 554 10.3389/fpsyg.2018.00554 29867619PMC5952033

# Response

**Published:** 2023-04-14

**Authors:** Yuji Ogihara

**Affiliations:** Psychology, Aoyama Gakuin Daigaku, Shibuya, Tokyo, Japan

Dear Dr. Han-Wu-Shuang Bao,

 Thank you very much for reviewing my manuscript and providing valuable comments.

 I have modified the manuscript according to your comments. I offer my responses to each comment below.

 I have copied and pasted all of your comments without making changes.

*In this article, the author summarized previous contradictory findings on the changes in individualism in China over the past decades, and provided further evidence from two social indicators (i.e., divorce rate and household size) for the increasing individualism in China. While data on such indicators are publicly available and quite easy to be collected from the National Bureau of Statistics of China (data.stats.gov.cn), it was indeed surprising that to date no studies have reported these indicators as evidence for cultural changes in China. Thus, the author’s current brief report would be necessary for a more complete understanding of Chinese cultural changes.*

*Nonetheless, I have some suggestions for the author to further improve this article, and also expect a revised version of the manuscript from the author.*

*1. The author collected the data in 2018, which covered the period only until 2017. This made the results somewhat out of date. Based on my own search (data.stats.gov.cn), it is certain that now the National Bureau of Statistics of China has updated the data until 2021. Therefore, I strongly suggest the author collecting more data points (i.e., 2018, 2019, 2020, and 2021) to provide the latest evidence. This would strengthen the potential impact of this article, and also help address the concern about the one-child policy because more data points (than merely 2016 and 2017) could be tested after the policy’s end in 2015 (i.e., to check whether household size continued to decrease even when Chinese parents are encouraged to give birth to more than one child since 2015).*

 I appreciate your constructive comment. I agree with you that including more data points is important in providing the latest evidence. Nevertheless, the four data points (2018, 2019, 2020, 2021) have not been newly added for three reasons.

 First, it is necessary to consider the complex effects of COVID-19 from 2019. This pandemic should have enormously affected Chinese culture and people. For example, the pandemic might have made Chinese people less individualistic. This hypothesis should be investigated in an independent study in detail, considering a series of previous studies regarding pandemics (e.g., Na et al., 2021; Schaller et al., 2022).

 Second, the main purpose of this article is to examine temporal changes in individualism in China. This purpose is already fulfilled in the current version of the article, which investigated changes over the past 65 years between 1953 and 2017. Again, I agree that adding the four data points would describe the latest situation, but describing the latest situation is not the main purpose of this article.

 Third, it is not always necessary to analyze the latest data when researchers publish academic articles. As you know, it takes time to publish academic articles. When I conducted this study, the data after 2017 were unavailable.

 However, if you think adding the four data points is a must, I will reconsider this point.

 Na, J., Kim, N., Suk, H. W., Choi, E., Choi, J. A., Kim, J. H., ... & Choi, I. (2021). Individualism-collectivism during the COVID-19 pandemic: A field study testing the pathogen stress hypothesis of individualism-collectivism in Korea. Personality and Individual Differences, 183, 111127. https://doi.org/10.1016/j.paid.2021.111127

 Schaller, M., Murray, D. R., & Hofer, M. K. (2022). The behavioural immune system and pandemic psychology: the evolved psychology of disease-avoidance and its implications for attitudes, behaviour, and public health during epidemic outbreaks. European Review of Social Psychology, 33, 360-396. https://doi.org/10.1080/10463283.2021.1988404

*2. It would be better to disclose the limitations of previous findings, which would help readers better evaluate different pieces of evidence. For example, in Santos et al.’s (2017) study, they only had 4 data points for China (1990, 1995, 2001, 2007) on three self-report items of cultural values ^1^. This could be main limitations of this specific finding and would make the idea that “individualism is decreasing in China” less convincing. The author may provide further critical comments when summarizing the literature.*

 Thank you for your helpful comment. Following your suggestion, I have added some explanations of the limitations of previous findings (Santos et al., 2017; Zeng & Greenfield, 2015) in Footnotes 1 and 2 as below.

 “Although this study described global trends of temporal changes in individualism, it has some limitations. For example, the authors aggregated three items from the World Values Survey to examine temporal shifts in individualistic values, but the inter-item correlations were not high (.11 < τ s < .24; ordinal-level Kendall’s τ ), possibly implying that the validity of these items may be low. Moreover, as the authors already noted in their article, the data points for China were only four (1990, 1995, 2001, and 2007), and they covered a relatively short period of time (17 years). This limited number of data points might have led to a failure to detect actual historical changes in individualistic values in China. These limitations may be related to the inconsistency of the findings. Indeed, their study showed that most (39 out of 53) of the countries they examined indicated a substantial increase in individualistic values, whereas only five countries (China, Armenia, Croatia, Ukraine, and Uruguay) exhibited a nonnegligible decrease in individualistic values.” (Footnote 1)

 “However, these words increased in frequency only slightly. As the authors emphasized in their article, the contrasting individualistic words (“choose” and “get”) that were analyzed increased more remarkably (for the details of their interpretation, see Zeng and Greenfield, 2015).” (Footnote 2)

*3. Since Bao et al. (2021) has provided more valid evidence for the increasing name uniqueness in China ^2^ and has addressed Ogihara’s (2020) ^3^ concerns on Cai et al. (2018) ^4^, it is necessary to also cite Bao et al. (2021) ^2^ when citing Cai et al. (2018) ^4^ in this article:*

*1. P. 2 (of the pdf version of the article) “Particularly, there has been only one study examining changes in the behavioral aspect in China (naming; Cai et al., 2018).” should be “Particularly, there has been two studies examining changes in the behavioral aspect in China (naming; Bao et al., 2021; Cai et al., 2018).”*

*2. P. 8 “Past research showed the rise in individualism in China (Bao et al., 2021; Cai et al., 2018; …)”.*

 I appreciate your suggestion. Actually, I already cited Bao et al. (2021) in the text (p. 3 in the PDF file) and the reference list in the previous version of the article, but I failed to cite the study in some points. Thus, following your comment, I have added the reference (Bao et al., 2021) in the two points in the new version of the article as you suggested.

 Thank you for your further consideration of this manuscript.

 I look forward to hearing from you at your earliest convenience.

 Sincerely,

 Yuji Ogihara, Ph.D.

 Department of Psychology, College of Education, Psychology and Human Studies, Aoyama Gakuin University

 Address: 4-4-25 Shibuya, Shibuya-ku, Tokyo, 150-8366, Japan

 E-mail: yogihara@ephs.aoyama.ac.jp

Web: https://sites.google.com/site/yujiogiharaweb/english
